# Differential Modulation of Attention by Aversive Associative and Statistical Learning in Distinct Visual Search Modes

**DOI:** 10.3390/bs15091274

**Published:** 2025-09-18

**Authors:** Yue Chen, Junzhen Guo, Chen Huang, Yingying Wang

**Affiliations:** Department of Psychology and Behavioral Sciences, Zhejiang University, 866 Yuhangtang Road, Hangzhou 310058, China; 22339049@zju.edu.cn (J.G.); 12139027@zju.edu.cn (C.H.); ywang15@zju.edu.cn (Y.W.)

**Keywords:** aversive associative learning, statistical learning, selection history, attention, visual search mode, proactive suppression

## Abstract

Selection history significantly influences attentional processes. Current debates center on whether different components of selection history influence attention through shared learning-dependent mechanisms or via independent mechanisms. Recent research suggests that aversive associative learning and statistical learning, two key components of selection history, modulate attentional selection independently. The present study investigates how these two components influence attentional selection under different search strategies. In Experiment 1, participants engaged in a singleton detection task, searching for a unique shape singleton while ignoring an irrelevant color singleton. In Experiment 2, they employed a feature search strategy, targeting a predefined attribute among varied shapes while disregarding a distracting color singleton. Results showed that under the singleton detection mode, two learning processes exert independent effects on attentional selection toward salient distractors. Conversely, under the feature search mode, the two learning processes interacted, with the interaction primarily driven by aversive associative learning. These findings highlight the critical role of search strategies in modulating how selection history affects attentional processes. They offer new insights into the mechanisms of attentional selection and the interplay between different forms of learning in complex visual search environments.

## 1. Introduction

The human visual system is often overwhelmed by a vast influx of information, with only a small subset deemed essential for further processing. Attention could prioritize specific sensory inputs for further analysis while concurrently suppressing irrelevant or potentially disruptive information (Carrasco, 2011; Chelazzi et al., 2019; Rusz et al., 2020). Recent research has revealed that such attentional guidance could be shaped by selection history—the accumulation of past search experiences that lead to biases in attentional selection (Anderson et al., 2021). Notably, selection history exhibits independent guidance on attention from the two primary attention modulation factors, top-down attentional goals and bottom-up stimulus salience (Awh et al., 2012; Luck et al., 2021). Given its distinct role, there is growing interest in understanding how selection history guides attention across different modes of visual processing.

Statistical learning and aversive associative learning are two forms of selection history that have been found to shape attentional prioritization (Jiang, 2018; Smith et al., 2006; Stankevich & Geng, 2014). Recent research distinguishes two main forms of statistical learning: object-based and location-based (Anderson et al., 2021). Object-based statistical learning involves learning the relationships and regularities among objects that recur across trials. For example, when a context has previously been associated with a specific stimulus in a memory task, presenting that context subsequently biases attention toward the corresponding stimulus (Nickel et al., 2020). In contrast, location-based statistical learning reflects the tendency to attend more to locations where targets or distractors frequently appear. For example, visual targets are detected more quickly and accurately when they appear in spatial locations that have been encountered more frequently in the past (e.g., Cohen & Magen, 1999; Geng & Behrmann, 2005; L. Huang & Pashler, 2005). Recently, research has increasingly focused on the role of location-based statistical learning in distractor suppression (Theeuwes et al., 2022). For instance, studies have shown that repeated exposure to distractors appearing in specific regions reduces the orienting reflex (B. Wang et al., 2019; Won et al., 2019) and induces proactive suppression to these locations (C. Huang et al., 2021, 2022; Kong et al., 2020).

Similarly, aversive associative learning can modulate attentional priority, making stimuli associated with aversive outcomes more attention-grabbing (Nissens et al., 2016). Previous studies in this area have often used electric shocks (Kim & Anderson, 2021; Ogden et al., 2023) or negative noise stimuli (Gutierrez & Berggren, 2019). However, regardless of the type of stimulus used, these studies have revealed that when distractors are paired with aversive stimuli, participants readily show a tendency to attend to them, likely to meet survival and adaptation needs (Gdalyahu et al., 2012; LeDoux, 1996). Together, these findings suggest that both statistical frequency and emotional value of past experiences play a crucial role in guiding attentional prioritization.

Despite the well-established role of statistical learning and aversive associative learning in guiding attention, it remains unclear whether these two forms of learning rely on shared attentional mechanisms or operate independently. To address this question, recent studies have manipulated statistical frequency and aversive value simultaneously on the same set of distractors, aiming to determine how these two components of learning mechanisms guide attention (Kim & Anderson, 2021; Ogden et al., 2023). If both components stem from a common mechanism that governs learning-based attentional control, their effects may be integrated within a unified attention biasing system, jointly biasing attention and competing for limited cognitive resources (Failing & Theeuwes, 2018; Theeuwes, 2019). Alternatively, if these components represent separate systems, they may modulate attention in parallel and independently. Empirical findings support the latter possibility. When distractors were simultaneously associated with aversive value and statistical frequency, the aversive value impaired the overall statistical learning effect but did not alter its pattern. Specifically, distractors carrying aversive value attracted greater attention, yet their attentional capture was reduced when they appeared in high-frequency locations compared to low-frequency locations. These findings suggest that aversive value and statistical frequency contribute to the attentional priority of distractors independently, indicating that these two components modulate attention through separate mechanisms.

Notably, evidence for such independent influences has primarily been derived from the additional singleton search paradigm, in which participants adopt a “singleton detection mode” (Gaspelin & Luck, 2018; Kerzel & Huynh Cong, 2024). This mode is thought to operate at the preattention stage, where search processes occur in parallel, and the attentional window is wide enough to encompass all items (Theeuwes, 1992, 2023). The independent contributions of aversive value and statistical frequency to distractor priority may reflect the parallel processing architecture of the singleton detection mode, where multiple learning mechanisms can bias attention simultaneously with no cognitive resource constraints (Gao & Theeuwes, 2022). In addition, although distractors at high-probability locations captured attention less than those at other locations, they still triggered substantial attention capture relative to the no-distractor condition, resulting in a phenomenon commonly known as singleton-presence cost (Kerzel et al., 2022; Zhao et al., 2024). This leads to the possibility that even though the aversive value and statistical frequency jointly modulate attention, their effects can be partially masked by the overall capture phenomenon.

In contrast, research on “feature search mode” suggests divergent results. In this mode, the attentional window narrows, allowing a serial search process. Participants focus on a specific target among heterogeneous items, which reduces or eliminates distractor interference (Bacon & Egeth, 1994; Gaspelin et al., 2015; Leber & Egeth, 2006; Ma & Abrams, 2023). Notably, recent studies indicate that in feature search mode, attention suppression is particularly pronounced at high-frequency distractor locations, which even eliminates the singleton-presence cost (Fan et al., 2021; Ivanov & Theeuwes, 2021; B. Wang & Theeuwes, 2018). Because learned distractor locations often induce location-based suppression, employing a feature search strategy to enhance this inhibitory effect may offer new insights into how statistical and aversive associative learning interact in shaping attentional control.

This study investigates whether aversive associative learning and statistical learning influence attention through a shared control mechanism or through independent mechanisms. To examine this, we elicited two distinct attentional modes across two experiments using controlled stimulus manipulations: a singleton detection mode in homogeneous arrays (Experiment 1) and a feature search mode in heterogeneous arrays (Experiment 2). In each experiment, statistical learning and aversive associative learning were independently manipulated. A one-phase design was implemented to allow for a more direct assessment of the relationship between these learning effects (Le Pelley et al., 2022). The interaction between statistical and aversive associative learning was analyzed within each condition.

## 2. Experiment 1

Experiment 1 employed a design similar to that of Le Pelley and colleagues (Le Pelley et al., 2022). We used an additional singleton task in which participants judged the spatial location of a spot within a shape singleton (the target) while ignoring the potential presence of a salient color singleton (the distractor). To examine the effect of statistical learning on feature-based attention, we manipulated the likelihood of the distractor appearing in different spatial locations, with probabilities varied from high (65.2%), low (8.7%), to rare (4.3%). To investigate the role of aversive associative learning, the distractor was assigned one of two potential singleton colors: one paired with an aversive noise (conditioned stimulus: CS+), and the other presented without any aversive noise (CS−) (Gutierrez & Berggren, 2019; Watson et al., 2019). We aimed to determine whether statistical learning and aversive associative learning influence the attention selection of the distractor independently or interactively by analyzing whether their effects showed an interaction or not.

### 2.1. Method

#### 2.1.1. Participants

Large (Cohen’s *d* = 0.70) and medium (Cohen’s *d* = 0.49) effect sizes have been reported for the influence of aversive stimuli (Gutierrez & Berggren, 2019) and statistical learning (Kerzel et al., 2022) on attention, respectively. We determined that at least 46 participants (Cohen’s *d* = 0.49) are needed to reach a power of 90% for a significance level of 0.05 (calculated by GPower3, Faul et al., 2007). A total of 48 participants (27 females, mean age = 18.85 years, *SD* = 1.27 years) were recruited from Zhejiang University, China. All participants had normal or corrected-to-normal vision. The study was approved by the ethics committee of the Department of Psychology and Behavioral Sciences at Zhejiang University.

#### 2.1.2. Apparatus

The experiment was conducted in a dimly lit room, with participants seated 65 cm from a gamma-corrected Cathode-Ray Tube (CRT) monitor (24 inches; 1920 × 1080 resolution; 100 Hz refresh rate). Stimuli were generated using MATLAB (https://www.mathworks.com/, The MathWorks, Natick, MA, USA) and the Psychtoolbox-3 (Brainard, 1997; Pelli, 1997).

#### 2.1.3. Stimuli and Procedure

**Figure 1:**
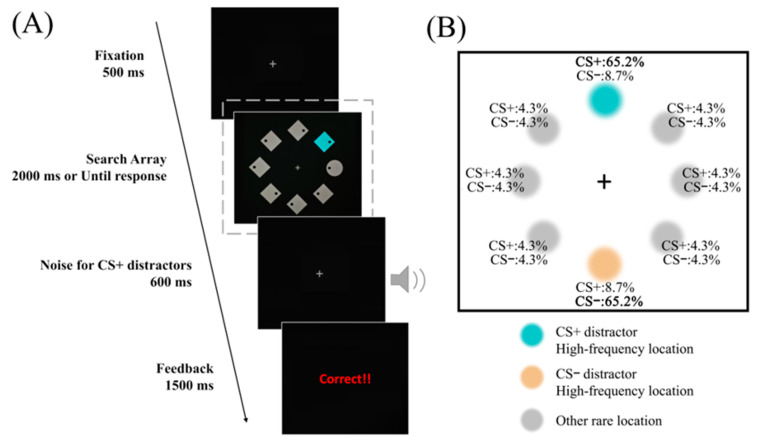
Procedure and design of Experiment 1. (**A**) Experimental procedure of Experiment 1. Participants identified the location of a dot inside a shape singleton (the target). The display sometimes included a color singleton (the distractor), which could be either orange or blue. When the CS+ distractor was present, an aversive sound would follow the response; when the CS− distractor was present, no aversive sound would be displayed. (**B**) The probability of distractor locations in Experiments 1 and 2. The CS+ high frequency location is represented by blue. Similarly, the CS− high frequency location is represented by orange. The CS+ and CS− distractors appeared at rare locations, represented by grey. Each location shows the probability of the distractors appearing there (when the distractor was present in the trials). CS+ and CS− distractors appeared most frequently at opposite locations. The frequent locations shown here are examples; these locations were chosen randomly for each participant.

As illustrated in Figure 1A, each trial began with a gray fixation cross (0.7° × 0.7°; [Red, Green, Blue] (RGB): [70, 70, 70]) displayed at the center of a dark gray screen (RGB: [5, 5, 5]) for 500 ms. This was followed by the stimulus display, which consisted of eight items (each occupying a luminous flux area of 5°) evenly spaced around an imaginary circle with a 5.7° radius, forming an octagonal arrangement.

Each display contained either one diamond among seven circles or one circle among seven diamonds, with the unique shape singleton serving as the target. In 82.1% of trials, a distractor featured with a salient color (blue: RGB [37, 141, 16] or orange: RGB [193, 95, 30]) was present, while in the remaining 17.9% of trials, no color singleton but only the shape singleton existed, serving as distractor-absent trials. For each participant, one singleton color was consistently paired with an aversive noise and designated as CS+, while the other singleton color was not paired with any noise and served as CS−. All other items were gray (RGB: [70, 70, 70]) (Le Pelley et al., 2022).

Across trials, each type of distractor (CS+ or CS−) appeared in one of the eight locations with varying probabilities (Figure 1B): one high-probability location (65.2%), one low-probability location (8.7%), and six rare-probability locations (4.3%). Following the design of Le Pelley et al. (2022), the high-probability location was randomly assigned for each participant, and the low-probability location was always directly opposite the high-probability location across the fixation cross. As a result, the two locations were always opposite to each other, and each served as the low-frequency position for the other. We ensured that these two locations were as different as possible and were equidistant from all other possible locations. This symmetrical arrangement avoided potential spatial deviations that may occur if key positions were adjacent or unevenly spaced in the display. In distractor-present trials, the target appeared randomized across all eight locations. In distractor-absent trials, the target appeared at the CS+ high-, CS− high-, and rare-probability locations with frequencies of 20%, 20%, and 10%, respectively. Notably, the high-probability location for CS+ always served as the low-probability location for CS−, and vice versa.

During the display of the stimulus array, a black dot (radius = 0.2°) was randomly positioned 0.8° to the left or right of the center of each item. Participants were instructed to identify the shape singleton (target) and indicate the dot’s location within the target as quickly and accurately as possible. Responses were made using a high-precision response box; participants pressed the left key with their left thumb for a black dot on the left, and the right key with their right thumb for a dot on the right. Reaction times (RTs) were recorded from the onset of the stimulus display. The display remained visible until the participant responded or until 2000 ms elapsed. When the display contained a CS+ distractor, an aversive noise (mean intensity = 54.41 dB, SD = 6.01 dB, the experimental materials are detailed in the database), sourced from the IADS-E database (Yang et al., 2018), was displayed for 600 ms immediately after stimulus offset. When the display contained a CS− distractor, a blank was displayed without noise for 600 ms (Watson et al., 2019). Feedback was then provided for the participant’s response.

Each participant completed eight experimental blocks, each consisting of 112 trials. The location probability manipulation followed Le Pelley et al.’s (2022) design. Within each block, there were 46 trials with a CS+ distractor, 46 with a CS− distractor, and 20 distractor-absent trials. The order of presentation was randomized within each block to ensure that participants could not predict the occurrence of distractor locations or CS+ and CS− trials. The assignment of the two colors to CS+/ CS− conditions was balanced across participants.

#### 2.1.4. Post-Experiment Measures

Following the experimental session, participants completed the Emotional Salience of Sounds Questionnaire B (12 items) to assess their emotional responses to the noise. They rated the acoustic characteristics of the noise experienced during the experiment (Masullo et al., 2021). If the score on the negative-emotions dimension exceeded that on the positive-emotions dimension, this indicated that the noise had a more negative emotional impact on the participants (as indicated in the Section 2.2.1).

Additionally, participants were shown a display without color distractors and were asked two questions to assess their awareness of statistical regularities in distractor locations (Corresponding to the Section 2.2.4): (1) “Was the probability of color distractors appearing in these eight locations the same or different?” (2) “In which specific location was the blue distractor more likely to appear, and in which specific location was the orange distractor more likely to appear?”

#### 2.1.5. Data Analysis

The attentional effect was calculated in both Experiment 1 and Experiment 2 by subtracting the RTs in the distractor-absent condition from those in each distractor-present experimental condition. A 2 (distractor value: CS+ vs. CS−) by 3 (location frequency: high, low, or rare) repeated measures Analysis of Variance (ANOVA) was performed to examine the main effect and interaction effect of the two components. Simple-effect analysis was performed with paired-sample t-tests. Bonferroni corrections were applied to correct for the multiple-comparison problem. We also reported (inverse) Bayes factors (BF01) to test any null results from the classical statistical tests. The BF01 indicates the strength of evidence in support of the null hypothesis, with values between 3 and 10 generally considered moderate evidence in favor of the null hypothesis (Kass & Raftery, 1995). We computed Bayes factors using a default prior in JASP 0.18.3.0.

### 2.2. Results

#### 2.2.1. Manipulation Checks

The Emotional Salience of Sounds Questionnaire B (Masullo et al., 2021) confirmed that participants perceived the noise as inducing more negative (*M* = 33.44) than positive (*M* = 21.48) emotions (*t*(47) = 8.32, *p* < 0.001, Cohen’s *d* = 1.21, *BF*_01_ = 8.56 × 10^−9^). This result demonstrates the effectiveness of negative emotions evoked by the noise.

#### 2.2.2. Target Detection in Distractor—Present Conditions

Error trials and trials with reaction times (RTs) below 200 ms, above 1500 ms, or exceeding three standard deviations from the mean were excluded, resulting in the removal of ~4.9% of the total observations. First, we verified that the detection of the targets was faster when the distractor was absent compared to when it was present. Results showed attentional capture by the distractors across the six conditions with varied distractor value (CS+ vs. CS−) and location frequency (high, low, or rare) (*p*s < 0.001).

**Figure 2:**
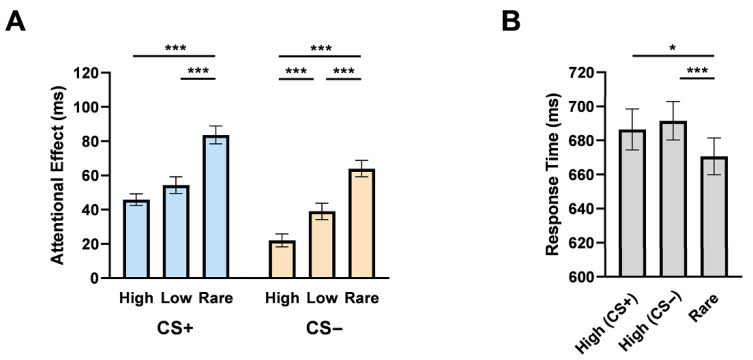
Results in Experiment 1. (**A**) The mean attentional effects in RTs. The attentional effects were increased in the CS+ condition than in the CS− condition and reduced in the higher frequency location. (**B**) Raw RTs of the target at three positions when distractors were absent. Error bars indicate *SEMs*. *, *p* < 0.05; ***, *p* < 0.001.

Next, we calculated the attentional effect by subtracting the RTs in the no-distractor condition from those in each distractor-present experimental condition. The attentional effects were submitted to a 2 (distractor value: CS+ vs. CS−) by 3 (location frequency: high, low, or rare) repeated measures ANOVA (Figure 2A). A significant main effect was found for distractor value (*F*(1,47) = 61.28, *p* < 0.001, *η_p_*^2^ = 0.57, *BF*_01_ = 3.72 × 10^−7^), revealing stronger attentional effects when the distractor was CS+ than CS−. Distractor location frequency also showed a significant main effect (*F*(2,94) = 77.18, *p* < 0.001, *η_p_*^2^ = 0.62, *BF*_01_ = 7.40 × 10^−18^), with attentional effects being reduced when distractors appeared in the higher frequency location. Importantly, consistent with Kim and Anderson (2021) and Zhao et al. (2024), the interaction effect between distractor value and location frequency was not significant (*F*(2, 94) = 1.03, *p* = 0.36, *η_p_*^2^ = 0.02, *BF*_01_ = 4.92).

Despite the nonsignificant interaction effect, we still performed a simple-effect analysis to explore the influence of statistical learning on distractors at different distractor values (Figure 2A). Results showed significant differences between every two of the location frequencies (*p*s < 0.001) in the attentional effects of the CS− distractor. The attentional effects also differed significantly between the CS+ rare and the CS+ high frequency location (*t*(47) = 8.40, *p* < 0.001, Cohen’s *d* = 1.21, *BF*_01_ = 6.63 × 10^−9^), or between the CS+ rare and the CS+ low frequency location (*t*(47) = 5.90, *p* < 0.001, Cohen’s *d* = 0.85, *BF*_01_ = 2.49 × 10^−5^). The attentional effect was numerically decreased in the CS+ high- compared to CS+ low frequency location (*t*(47) = −2.10, *p* = 0.12, Cohen’s *d* = 0.30, *BF*_01_ = 0.86). Overall, the attentional distribution was sensitive to the two levels of distractor value and three levels of statistical frequency.

The same repeated-measures ANOVA was applied to the error rate results, which showed a significant main effect of location frequency (*F*(2,94) = 4.01, *p* = 0.021, *η_p_*^2^ = 0.08, *BF*_01_ = 0.67), with higher error rates for high frequency locations (2.23%) than low frequency locations (1.86%). The main effect of distractor value (*F*(1,47) = 0.90, *p* = 0.35, *η_p_*^2^ = 0.02, *BF*_01_ = 7.03) and the interaction of the two effects (*F*(2,94) = 1.61, *p* = 0.21, *η_p_*^2^ = 0.03, *BF*_01_ = 3.11) were not significant.

#### 2.2.3. Target Location Effects When Distractors Are Absent

To further confirm that suppression of attention had been developed at the frequent locations of the distractor, we extracted the RTs for target detection in the distractor-absent trials. We conducted a repeated measures ANOVA to explore whether attention was suppressed when the target appeared at the high frequency (i.e., CS+ high and CS− high) than at rare frequency locations (Figure 2B). A significant main effect of different frequency locations was found (*F*(2,94) = 6.44, *p* = 0.002, *η_p_*^2^ = 0.12, *BF*_01_ = 0.08). Specifically, detection for targets appeared at rare locations were significantly faster than that at the CS+ high location (*t*(47) = 2.99, *p* = 0.013, Cohen’s *d* = 0.43, *BF*_01_ = 0.13) and CS− high location (*t*(47) = 3.98, *p* < 0.001, Cohen’s *d* = 0.57, *BF*_01_ = 0.01), showing that participants initiated suppression on attention to the locations that distractors were highly likely to appear. There was no significant difference in RTs between the target at the CS+ and CS− high location (*t*(47) = 0.68, *p* = 1.00, Cohen’s *d* = 0.10, *BF*_01_ = 5.11), suggesting a similar influence of emotional values on attention suppression. No significant effects were found in error rates (*p*s > 0.76).

#### 2.2.4. Explicit Recognition of Statistical Regularities

We assessed participants’ ability to explicitly identify the high and low frequency locations. Participants who correctly identified the high-frequency location (N = 9) or partially recognized that a certain location was with higher frequency (N = 3) were excluded from analysis. Among the remaining 36 participants, the proportion of incorrect responses was significantly greater than chance level (*p* < 0.001, *BF*_01_ = 0.003), indicating a lack of explicit recognition of the statistical regularities. We found that the attentional results were not changed even among these participants. Specifically, the 2 (distractor value: CS+ vs. CS−) by 3 (location frequency: high, low, or rare) repeated measures ANOVA still showed significant main effects of distractor value (*F*(1,35) = 45.98, *p* < 0.001, *η_p_*^2^ = 0.57, *BF*_01_ = 7.75 × 10^−6^) and location frequency (*F*(2,70) = 58.12, *p* < 0.001, *η_p_*^2^ = 063, *BF*_01_ = 2.10 × 10^−13^). Finally, there was no significant interaction between the distractor value and location frequency (*F*(2,70) = 1.42, *p* = 0.25, *η_p_*^2^ = 0.04, *BF*_01_ = 3.55).

### 2.3. Discussion

Experiment 1 confirmed that both aversive associative learning and statistical learning modulate attention to distractor locations. Specifically, distractors paired with aversive noise (CS+) caused greater response delays than neutral distractors (CS−), suggesting that aversive conditioning increased distractor salience and diverted attention from the target effectively (Gdalyahu et al., 2012; Schmidt et al., 2015). In contrast, the attentional modulation effect of statistical learning was reflected in significant proactive suppression in high-frequency conditions. This was evidenced by a reduced interference effect for distractors appearing in locations with higher frequency, as well as the slower response times to target items in higher frequency locations when distractors were absent. These results indicate that the spatial probability distribution of distractors may alter priority computations in attentional selection (Awh et al., 2012; Ferrante et al., 2018).

Importantly, the one-phase experimental design used in this study demonstrated that aversive associative learning and statistical learning independently influenced attentional allocation. A Bayesian analysis provided moderate evidence that these effects do not interact. Our findings here align with previous research on the impact of positive stimuli on attention and further extend these effects to aversive stimuli (Kim & Anderson, 2019, 2020, 2021; Le Pelley et al., 2022). This might be because the attentional system may be more strongly influenced by the motivational salience of stimuli—whether positive or negative—rather than their specific valence (Watson et al., 2019).

## 3. Experiment 2

Experiment 2 explored whether the interaction between statistical learning and aversive associative learning would change when a feature search mode was recruited in attentional guidance. In contrast to the broader attentional window engaged during singleton detection, feature search typically involves a narrower attentional focus (Bacon & Egeth, 1994; Theeuwes, 2023), which may reduce attention capture by distractors, potentially by enhancing proactive suppression of distractor locations (Gaspelin et al., 2015; Ma & Abrams, 2023), to conserve cognitive resources for goal-relevant target detection. However, due to the adaptive importance of threat-related information (LeDoux, 1996, 2014; Y. Wang et al., 2023), aversive associative learning may still capture attention, particularly when the aversive signal is with high spatial stability. We therefore hypothesized that aversive associative learning may modulate the attentional suppression induced by statistical learning, leading to an interaction between these two learning mechanisms.

### 3.1. Method

#### 3.1.1. Participants

A new set of 48 participants (22 females, 26 males; mean age = 18.96 years, *SD* = 1.05) was recruited from Zhejiang University. The same recruiting criteria were used as in Experiment 1.

#### 3.1.2. Stimuli and Procedure

**Figure 3:**
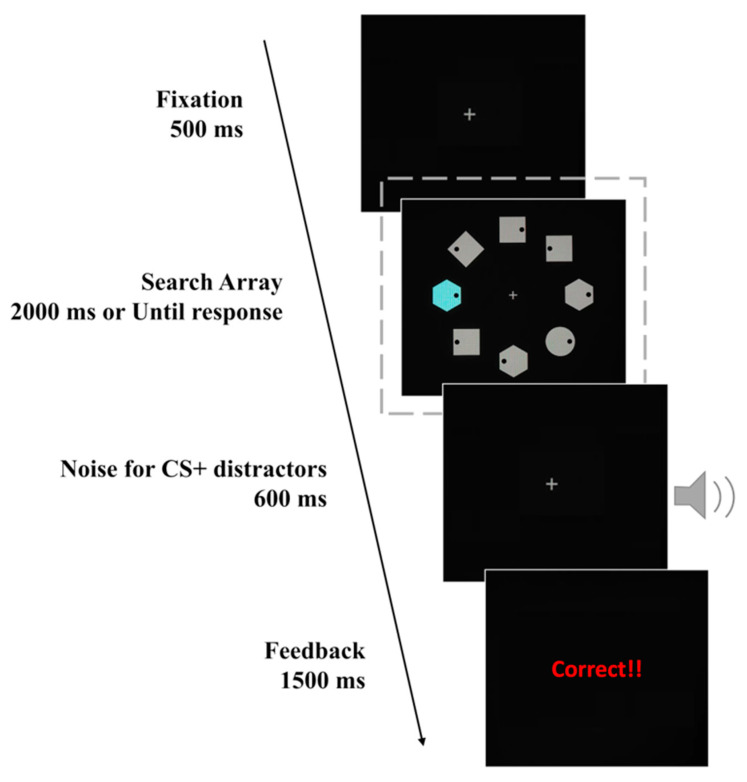
Procedure of Experiment 2. Participants responded to the location of the dot within a preassigned specific shape, rather than to a singleton.

The procedure of Experiment 2 closely followed that in Experiment 1, with identical manipulations for aversive associative learning and statistical learning. The key difference was in the composition of the search array. Specifically, the search arrays were modified to encourage participants to focus on a specific shape, as opposed to searching for a singleton. As shown in Figure 3, each search array consisted of four shape types: one circle, one diamond, three squares, and three hexagons. Participants were tasked with identifying a pre-specified shape (the target) and reporting the position of a black dot within this target. For half of the participants, the target was always the diamond, while for the other half, the target was the circle. Unlike the target, the distractor was not tied to a specific shape but instead to a spatial location. The assignment and frequency of distractor locations remained consistent with Experiment 1 (see Figure 1B).

### 3.2. Results

#### 3.2.1. Manipulation Checks

Post-experiment questionnaire showed that participants reporting more negative (*M* = 33.02) than positive (*M* = 21.79) feelings (negative vs. positive score: *t*(47) = 8.54, *p* < 0.001, Cohen’s *d* = 1.25, *BF*_01_ = 4.12 × 10^−9^) to the noise.

#### 3.2.2. Target Detection in Distractor—Present Conditions

Data cleansing was performed before formal statistical analysis, which excluded ~4.5% of the observations from the experiment. We first examined whether the detection of the targets was faster when the distractor was absent compared to when it was present. In vast contrast to Experiment 1, results showed that attentional capture by the distractor appeared only in the CS+ high frequency location (*t*(47) = 3.14, *p* = 0.003, Cohen’s *d* = 0.45, *BF*_01_ = 0.09) and the CS+ rare location (*t*(47) = 5.24, *p* < 0.001, Cohen’s *d* = 0.76, *BF*_01_ = 2.04 × 10^−4^). The attention effects of other conditions, including the CS+ low frequency location and all the CS− conditions, were not significant (*p*s > 0.17).

**Figure 4:**
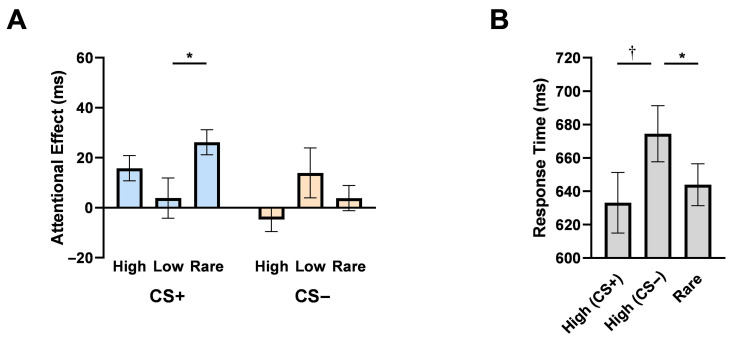
Results in Experiment 2. (**A**) The mean attentional effects in RTs. The attentional effects were increased in the CS+ condition than in the CS− condition. The effects interacted between the distractor value and location frequency. (**B**) Raw RTs of the target at three positions when distractors were absent. Error bars indicate *SEMs*. †, *p* < 0.09; *, *p* < 0.05.

As in Experiment 1, a 2 (distractor value: CS+ vs. CS−) by 3 (location frequency: high, low, or rare) repeated-measures ANOVA was performed on the attentional effect in RTs (Figure 4A). A significant main effect of distractor value (*F*(1,47) = 6.76, *p* = 0.01, *η_p_*^2^ = 0.13, *BF*_01_ = 0.81), indicating successful associative learning. However, the main effect of location frequency was not significant (*F*(2,94) = 1.07, *p* = 0.35, *η_p_*^2^ = 0.22, *BF*_01_ = 6.94). Interestingly, in contrast to Experiment 1, the distractor value showed a significant interaction with location frequency (*F*(2,94) = 5.71, *p* = 0.005, *η_p_*^2^ = 0.11, *BF*_01_ = 0.05). We further examined the different attentional effects of statistical learning in the CS+ and CS− conditions, separately. Simple-effect analysis showed that, for CS+ distractors, the attentional effect in the low-frequency condition was significantly lower than that in the rare frequency condition (*t*(47) = 2.68, *p* = 0.03, Cohen’s *d* = 0.39, *BF*_01_ = 0.27), indicating that participants could avoid attention distraction at the CS+ low condition. However, the attentional effect for CS+ high condition was no longer significantly lower than that for CS+ low (*t*(47) = 1.32, *p* = 0.58, Cohen’s *d* = 0.19, *BF*_01_ = 2.84) or CS+ rare (*t*(47) = −1.65, *p* = 0.32, Cohen’s *d* = 0.24, *BF*_01_ = 1.82) condition, showing failed avoidance of distractors when emotionally aversive distractors exhibited high spatial stability. For the CS− distractors, no significant difference was observed between two of the three frequency conditions (*p*s > 0.26), which aligned with the non-significant attentional effects in any of the three conditions. There were no significant main effects or interaction effects on error rates (*p*s > 0.12).

#### 3.2.3. Target Location Effects When Distractors Are Absent

To further confirm the location-specific attentional suppression, we analyzed target detection performance in distractor-absent trials using a repeated-measures ANOVA (Figure 4B). Results showed a significant main effect of location frequency (*F*(2,94) = 4.50, *p* = 0.01, *η_p_*^2^ = 0.87, *BF*_01_ = 0.37). Specifically, detection for targets at rare locations was significantly faster than that at the CS− high location (*t*(47) = 2.66, *p* = 0.03, Cohen’s *d* = 0.38, *BF*_01_ = 0.28), showing attentional suppression at the distractor-associated location in the CS− condition. In contrast, such attention suppression no longer existed in the CS+ high condition. In fact, responses were marginally significantly faster when the target appeared at the CS+ high location compared to the CS− high location (CS− high vs. CS+ high: *t*(47) = 2.28, *p* = 0.08, Cohen’s *d* = 0.33, *BF*_01_ = 0.62). Responses to targets at the CS+ high location were not significantly slower than those to the rare location (rare vs. CS+ high: *t*(47) = 0.87, *p* = 1.00, Cohen’s *d* = 0.13, *BF*_01_ = 4.45). These results suggest that attentional priority may be preserved at spatial locations consistently associated with aversive emotional value, effectively counteracting suppression effects seen with statistical regularities alone. No significant results were found in error rates (*p*s > 0.09).

#### 3.2.4. Explicit Recognition of Statistical Regularities

We excluded 13 participants who had at least partially identified the high- and low-frequency locations. The proportion of incorrect choices for the remaining 35 participants was significantly greater than chance level (*p* = 0.002, *BF*_01_ =0.034). A 2 (distractor value: CS+ vs. CS−) by 3 (location frequency: high, low, or rare) repeated-measures ANOVA showed that the result pattern was not altered. Specifically, a significant main effect for distractor value (*F*(1,34) = 14.20, *p* = 0.001, *η_p_*^2^ = 0.30, *BF*_01_ = 0.11) was found. The main effect for location frequency was still not significant (*F*(2,68) = 0.57, *p* = 0.57, *η_p_*^2^ = 0.02, *BF*_01_ = 7.60). Importantly, the interaction effect between distractor value and location frequency was still significant (*F*(2,68) = 4.87, *p* = 0.01, *η_p_*^2^ = 0.13, *BF*_01_ = 0.11).

#### 3.2.5. Comparison Between the Attentional Effects in Experiments 1 and 2

**Figure 5:**
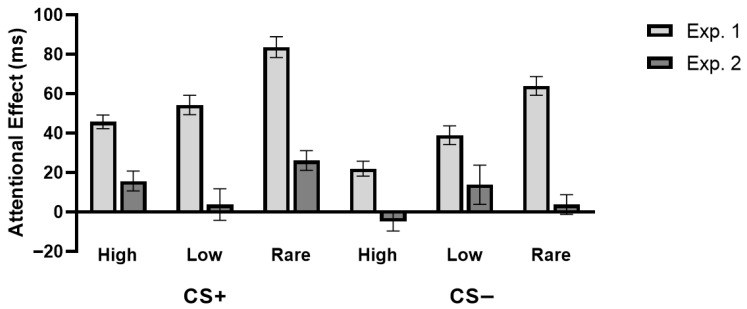
The attentional effects in Experiment 1 (Exp. 1) and Experiment 2 (Exp. 2). Grey bars indicate the mean RTs, and error bars indicate *SEMs*.

Finally, we compared the attentional effects in Experiments 1 and 2 (Figure 5). A 2 (distractor value: CS+ vs. CS−) by 3 (location frequency: high, low, or rare) by 2 (experiment: Experiment 1 vs. Experiment 2) mixed-effect ANOVA was performed on the attentional effect in RTs. Confirming that the attentional effect is weakened in feature search mode, results revealed that the attentional effect was smaller in Experiment 2 (mean = 9.81, *SD* = 46.51) compared to Experiment 1 (mean = 51.49, *SD* = 36.70) (main effect of experiment: *F*(1, 94) = 62.35, *p* < 0.001, *η_p_*^2^ = 0.40, *BF*_01_ = 3.39 × 10^−4^). Critically, the interaction effect across the three factors was marginally significant (*F*(2, 188) = 2.88, *p* = 0.059, *η_p_*^2^ = 0.03, *BF*_01_ = 0.96), suggesting that aversive associative learning and statistical learning produced different attentional effects in the two experiments.

To further evaluate the attentional effects due to aversive associative learning and statistical learning, we calculated the relative attentional effects between high and low frequency conditions relative to the rare frequency condition, separately for the CS+ and CS− conditions in each experiment. For the CS− condition, the interaction between the location frequency and experiment was not significant (*F*(1, 94) =0.02, *p* = 0.89, *η_p_*^2^ < 0.001, *BF*_01_ = 4.39), with lower attentional effects observed in both CS− high (*t*(94) = −5.44, *p* < 0.001, Cohen’s *d* = 0.56, *BF*_01_ = 3.28 × 10^−5^) and CS− low (*t*(94) = −3.23, *p* = 0.002, Cohen’s *d* = 0.33, *BF*_01_ = 0.05) locations in Experiment 2 than Experiment 1.

For the CS+ condition, the interaction between the location frequency and experiment was significant (*F*(1, 94) = 4.24, *p* = 0.04, *η_p_*^2^ = 0.04, *BF*_01_ = 0.73). Further analysis showed that the relative attentional effect of CS+ distractors at high-frequency locations was significantly reduced in the feature search mode (Experiment 2) compared to the singleton detection mode (Experiment 1) (*t*(94) = −3.55, *p* = 0.001, Cohen’s *d* = 0.36, *BF*_01_ = 0.02). In contrast, for CS+ distractors at low-frequency locations, attentional effects did not differ between the two experiments (*t*(94) = −0.73, *p* = 0.47, Cohen’s *d* = 0.07, *BF*_01_ = 3.68). Therefore, the different attention modulation in the two visual search modes was mainly driven by the learnt aversive associations.

### 3.3. Discussion

In contrast to Experiment 1, Experiment 2 revealed a significant interaction between statistical learning and aversive associative learning, indicating that when attention is guided by a feature search mode, the influence of these two learning mechanisms on attentional allocation becomes interdependent. A direct comparison across the two experiments identified that the influence of distinct visual detection modes primarily emerged in the CS+ condition. Specifically, for the CS+ and CS− conditions in Experiment 1, statistical learning led to greater proactive suppression at high-frequency distractor locations than low-frequency distractor locations—consistent with typical statistical learning effects. However, this pattern reversed in the CS+ condition of Experiment 2, where proactive suppression was reduced for CS+ high-frequency distractor locations. Supporting this observation, reaction time (RT) data from distractor-absent trials also showed a failure to suppress responses at the CS+ high-frequency location (see Figure 4B), mirroring the pattern seen in distractor-present trials.

Under conditions of increased cognitive demands, as in the feature search task (Theeuwes, 2023) in Experiment 2, participants initiated stronger proactive suppression of distractor locations in order to complete the central task (as shown by evidence in the CS− condition). However, in contexts where aversive stimuli exist, proactive suppression may be reduced to these high-value locations as a precautionary measure. Given the evolutionary importance of threat detection (LeDoux, 1996, 2014), CS+ high-frequency locations—being the most consistently associated with danger in our study—may have acquired elevated attentional priority and reduced suppression in order to facilitate responses to potential harm. These findings align with recent research, demonstrating that both reward- and punishment-based learning can shape spatial priority maps (Anderson et al., 2022; Chelazzi et al., 2014).

## 4. General Discussion

This study examined how aversive associative learning and statistical learning affect attentional allocation under different visual search modes. We found that target detection was improved when distractors appeared in higher-frequency locations, supporting individuals’ ability to learn to suppress attention to frequently occurring distractor locations (Ferrante et al., 2018; Theeuwes et al., 2022). Additionally, distractors associated with aversive emotional values (CS+ distractor) captured more attention than neutral distractors (CS−), suggesting that aversive stimuli can enhance attentional prioritization (Gdalyahu et al., 2012; Schmidt et al., 2015; Stankevich & Geng, 2014).

Experiment 1 replicated the findings of Kim and Anderson (2021), demonstrating that aversive associative learning and statistical learning independently influence attention in the singleton detection mode. Kim’s two-stage study provided evidence that associative learning (of both reward and aversive stimuli) and statistical learning modulate attention separately. This conclusion was later supported by Le Pelley et al. (2022), who used a one-phase design to reduce confounds and improve reliability. Following this approach, our study replaced reward-based associative learning with aversive associative learning. Consistent with prior work, our results confirmed the independence of the two learning processes. Notably, given that the associative learning of reward and aversive stimuli results in similar results, it is likely that attentional orienting is driven more by motivational salience than by the valence of stimuli. Supporting this, neuroimaging studies (Sokol-Hessner et al., 2013) have shown similar amygdala activations to cues predicting rewards and punishments. Likewise, ERP findings (Brosch et al., 2008) indicate that both fear-related and nurturance-related stimuli evoke similar early neural responses. These results suggest that attentional selection is guided by the relevance of stimuli for survival, rather than their positive or negative valence.

In Experiment 2, we found that aversive associative learning and statistical learning jointly influenced attention in the feature search mode. This is in contrast with the independent attentional effects observed in the singleton detection mode in Experiment 1. Notably, the singleton detection mode primarily engages bottom-up attentional processes, which allows for automatic attention capture driven by physical saliency (Theeuwes, 1992). In this context, both aversive value and statistical frequency can serve as sources of physical salience (Chelazzi et al., 2014). Supporting this interpretation, neuroimaging studies have demonstrated that attention capture by aversive and statistical learning involves distinct neural substrates in the singleton detection mode: statistical learning engages the medial temporal lobe and hippocampus (Chun & Phelps, 1999; Durran et al., 2013), whereas aversive stimuli engage the amygdala and visual cortex (Lim et al., 2009). By comparison, the feature search mode in Experiment 2 relies more heavily on top-down control mechanisms, and the top-down goal-directed attentional strategies may override stimulus-driven salience (Gaspelin et al., 2015; Stilwell et al., 2024). Evidence has shown that increased task demands in feature search narrow the attention window and modulate brain networks in a complex manner (Gerchen & Kirsch, 2017). In this context, the prefrontal cortex is likely to play a critical role in modulating the attentional control process and integrating higher-level information (McNab et al., 2008; Minamoto et al., 2010). These top-down processes may support the observed interaction between aversive associative learning and statistical learning in Experiment 2.

It has long been suggested that statistical learning is an automatic learning process (Chun & Jiang, 1998; Theeuwes, 2025; Turk-Browne et al., 2005). In this study, we investigated whether statistical learning could operate in the absence of explicit knowledge. We employed an offline test asking participants to recognize the locations associated with different distractor frequencies. Even after excluding participants who identified the high- and low-frequency distractor locations, we still observed the independent and interactive effects between the two learning processes in the two experiments. Notably, our subjective test was vulnerable to conservative response biases (Vicente-Conesa et al., 2023), and thus could not definitively rule out all conscious contributions (Vadillo et al., 2016; Le Pelley et al., 2022). Therefore, we only suggest that the observed attentional inhibition was not driven by goal-directed control based on explicit knowledge. Instead, the effects are more consistent with attentional modulation driven by selection history (e.g., Ferrante et al., 2018; B. Wang & Theeuwes, 2018).

Future studies should pair our paradigm with more sensitive awareness measures that could disentangle genuine unconscious learning from response criterion effects to explore whether the interaction between the two learning processes occurs automatically and unconsciously. In addition, we used relatively mild aversive stimuli (noises) rather than electric shocks to investigate whether aversive stimulus modulates the attentional effects. Although previous studies did not observe differences in attentional guidance between the two types of negative stimuli, this issue warrants further investigation. Finally, our work primarily focused on location-based statistical learning, leaving the relationship between object-based learning and aversive learning still unresolved. This issue could be considered in future research.

In summary, this study employed a one-phase design to investigate how aversive associative learning and statistical learning influence attentional guidance across different visual search strategies. Experiment 1 showed that under singleton detection mode, the two learning mechanisms exerted independent effects on attention. Experiment 2 revealed that under feature search mode, aversive and statistical learning interacted to jointly modulate attentional allocation. These findings underscore the adaptability of the attention system in adapting to complex and dynamic environments by integrating multiple learning signals.

## Data Availability

Data from this study are available at https://osf.io/vjn5k/ (accessed on 19 August 2025).

## Abbreviations

The following abbreviations are used in this manuscript:

CRT	Cathode-Ray Tube
RGB	Red, Green, Blue
ANOVA	Analysis of Variance
CS+	Conditioned Stimulus, a stimulus presented with an aversive noise
CS−	The stimulus was presented without any aversive noise
Exp. 1	Experiment 1
Exp. 2	Experiment 2
